# Effects of microbes in pig farms on occupational exposed persons and the environment

**DOI:** 10.1186/s13568-023-01631-x

**Published:** 2023-11-30

**Authors:** Jinyi Han, Mengyu Li, Xin Li, Chuang Liu, Xiu-Ling Li, Kejun Wang, Ruimin Qiao, Feng Yang, Xuelei Han, Xin-Jian Li

**Affiliations:** 1 College of Animal Science and Technology, Henan Agricultural University, Zhengzhou, 450002 China; 2 Sanya Institute, Hainan Academy of Agricultural Science, Sanya, China

**Keywords:** Pig farming, 16S rRNA, Occupational exposure, Dust microbes, Microbial aerosols

## Abstract

In terms of pig farming, pig gut microbes have a significant effect on farmers and the farm environment. However, it is still unclear which microbial composition is more likely to contribute to this effect. This study collected a total of 136 samples, including pigs’ faeces samples, farmers’ faeces samples, samples from individuals who had no contact with any type of farm animal (referred to as ‘non-exposed’ persons), and environmental dust samples (collected from inside and outside pig houses and the farm) from two pig farms, pig farm A and pig farm B. Whereafter, 16S rRNA sequencing and taxonomic composition analysis were performed. According to the study, compared to non-exposed persons, pig farmers had a significantly higher abundance of 7 genera. In addition, the farmers were grouped according to the duration of their occupational exposure, and it was shown that 4 genera, including *Turicibacter*, *Terrisporobacter*, and *Clostridium_sensu_stricto_1*, exhibited a rise in more frequent contact with pigs. As compared to outside the pig house, the environmental dust has a greater concentration of the 3 bacteria mentioned before. Therefore, these 3 microbes can be considered as co-occurring microbes that may exist both in humans and the environment. Also, the 3 co-occurring microbes are involved in the fermentation and production of short-chain fatty acids and their effectiveness decreased as distance from the farm increased. This study shows that the 3 microbes where pig farmers co-occur with the environment come from pig farms, which provides fresh ideas for preventing the spread of microbial aerosols in pig farms and reducing pollution.

**Supplementary Information:**

The online version contains supplementary material available at 10.1186/s13568-023-01631-x.

## Introduction

Pigs are the dominant species in livestock production, and there is a resemblance between the gut microbiomes of pigs and humans. This is because around 96% of the functional pathways identified in the human gut microbiome are also present in pigs (Xiao et al. 2016; Patil et al. 2020). However, pig farmers work in an environment that is rich in pig-related microbes. Prolonged occupational exposure can potentially impact an individual's microbiota. Consequently, this sets them apart from individuals who are not occupationally exposed to pigs (Hong et al. 2021; Sun et al. 2020). Studies indicate that individuals working on pig farms may experience notable changes in their nasal microbes, resulting in a significantly more diverse nasal microbiota (Kraemer et al. 2019, 2021). The pig farm environment also has an impact on the composition of faeces microbes in pig farm workers (Sun et al. 2017). It is possible that the process of aerosolization, where pig faeces microbes from the upper respiratory tract enter the gastrointestinal tract, could result in changes to the population of faeces microbes (Moor et al. 2021). Many factors, including dietary habits and the environment, have an impact on the composition of the gut microbes, which in turn influences physical health in a variety of ways (Shang et al. 2022; Martel et al. 2022; Yang et al. 2022). Therefore, it is crucial to investigate the effects of occupational exposure on the intestinal microbes of farmers.

The atomization of pig faeces microbes can also facilitate the exchange of microorganisms in the air both inside and outside the pig house, leading to changes in the environmental microbes of the pig farm (Hong et al. 2021; Luiken et al. 2020). For instance, the odor emanating from the pig farm is primarily caused by *Clostridium* (Zhu 2000). And within the pig farm, there is a significant correlation between dust and *Staphylococcus aureus* exposure in farms (Madsen et al. 2019). Both fungi and anaerobic bacteria have the potential to create poor indoor air quality, which can increase the chances of pigs getting sick (Kristiansen et al. 2012). In addition, pig faeces microbes become dust microbes through aerosol transmission, which will change antibiotic resistance and the composition of microbial communities in soil (Gao et al. 2020a; Van Gompel et al. 2020; Luiken et al. 2020). However, it is worth noting that farms generally exhibit higher levels of microbial diversity. When farmers bring these dust microbes home, the indoor microbiota can resemble that of a farm and play a crucial role in protecting children from developing asthma (Kirjavainen et al. 2019; Wang et al. 2023). Therefore, the influence of pig farms on microorganisms in the surrounding environment has long been a research hotspot. In order to facilitate subsequent analysis and improve microbial contamination of pig faeces, we hope to explore the common susceptible microbes in pigs, humans and the environment based on the concept of “one health”. The study aims to provide theoretical support for reducing pollution and preventing the spread of aerosol of microorganisms in pig farms. Also, it will demonstrate the sustainable development of pig farms from a microorganism perspective.

Two pig farms were sampled and analyzed for the study, pig farm A and pig farm B. The first focus of the study was on the differences in faecal microorganisms between pig farmers and non-exposed persons. Additionally, the study inferred the path and distance of microbial diffusion by analyzing the distribution of dust microbes in the pig farm environment.

## Material and methods

### Sample collection

In this study, two pig farms were sampled, pig farm A and pig farm B in different provinces of China. Primarily, a total of 136 samples were gathered from pig farm A in December 2021, including 22 samples of human faeces, 70 samples of pig faeces, and 44 environmental samples. Out of the 22 samples of human faeces, 18 were obtained from farmers (13 breeders and 5 less exposed individuals), while the remaining 4 were from non-exposed persons who had no contact with farm animals. A total of 44 environmental samples were gathered from the pig house’s interior, outside, living area, and cesspool, among other places (see Table 1). 3 particular areas within the pig house were sampled: the floor, the handrail, and the inside windscreen, a deflector plate that controls the airflow within the pig house (see Additional file 1: Fig. S1). Each location was sampled 4 times. 5 samples were taken at each of 3 points outside the farm that were 5, 500, and 1000 m from the pig farm, respectively.

**Table 1:** The specific sampling plan and number

Group	Location	Number of samples from pig farm A	Number of samples from pig farm B
Pig faeces	Pig	70	10
Dust inside the pig house	Handrail	4	–
	Floor	4	4
	Indoor windscreen	4	4
Dust outside the pig house	Living	4	–
	Outdoor windscreen	4	4
	Outside	3	–
	Cesspool	6	–
Dust outside the pig farm	5 m outside the farm	5	4
	500 m outside the farm	5	4
	1000 m outside the farm	5	4
Volunteer’s faeces	Breeder	13	4
	Leader	5	3
	Non-exposure	4	2

In November of the subsequent year, samples were collected from pig farm B. A total of 43 samples were obtained, including 10 pig faeces samples, 24 environmental samples, and 9 human faeces samples. The 9 human faeces samples were obtained from 7 farmers, including 4 breeders and 3 less exposed leaders. It is worth mentioning that two of breeders used to be the leaders of pig farm A for microbial collection, but they had been out of work for half a year and had not been exposed to animals for three consecutive months at the time of this sampling. A total of 24 environmental samples were collected from both the inside and outside of the pig house. 3 places were selected in the pig house: the floor, the windscreen outside the pig house, and the windscreen inside the pig house. 4 samples were obtained at each of 3 points in the area outside the house, which were spaced apart by 5, 500, and 1000 m. The specific sampling plan and number are shown in Table 1.

Fresh faeces samples were collected from pigs using rectal palpation. Each pig was sampled using a new nitrile examination glove. After that, the samples were squeezed into frozen tubes. The volunteers collected their own faeces samples, which were subsequently placed in frozen tubes and stored in liquid nitrogen. Environmental dust samples were collected using sterile swabs and transferred into sterile centrifuge tubes. The samples were placed in liquid nitrogen immediately after collection. All samples were promptly transported to the liquid nitrogen in the laboratory and stored at − 80 °C until further analysis.

### DNA extraction and polymerase chain reaction (PCR) amplification

DNA was extracted using the OMEGA Soil DNA Kit (Omega Bio-Tek, Norcross, GA, USA) and stored at – 20 °C before analysis. From these DNA extracts, the V3–V4 region of the 16S rRNA gene was amplified using forward (5′-ACTCCTACGGGAGGCAGCA-3′) and reverse (5′-GGACTACHVGGGTWTCTAAT-3′) primers, and Illumina linker sequences were used at 5′ end. PCR cycling conditions included initial denaturation at 95 ℃ for 5 min and 25 cycles of denaturation at 95 ℃ for 30 s, annealing at 59 ℃ for 30 s and extension at 72 ℃ for 45 s. This process was followed by a final extension step at 72 °C for 5 min. PCR amplicons were purified using Vazyme VAHTS™ DNA Clean Beads (Vazyme, Nanjing, China) and quantified using the Quant-iT PicoGreen dsDNA Assay Kit (Invitrogen, Carlsbad, CA, USA). The subsequently constructed library was sequenced on the Illumina HiSeq 2500 platform (2 × 250 pairs), and sequencing was performed by Shanghai Personal Biotechnology Co., Ltd. (Shanghai, China).

### 16S rRNA gene sequence assembly and clustering

16S rRNA sequencing data were processed using the Quantitative Insight Into Microbial Ecology 2 (QIIME2, 20019.4) platform (Bolyen et al. 2019). Then, the sequences were quality filtered, denoised, and merged, and chimeras were removed using the DADA2 plugin (Callahan et al. 2016). Non-single amplicon sequence variants (ASVs) were aligned with mafft (Katoh et al. 2002). Subsequently, species taxonomic annotation was performed using the SILVA database (Release132, http://www.arb-silva.de), and the classify-sklean algorithm with default parameters was used in the analysis in QIIME2. Moreover, each naive Bayes classifier was used to obtain the composition and abundance of individual samples in the taxonomic-level distribution table. The resulting ASV scale was flattened with QIIME2 using rarefaction at the 95% depth of the lowest sample sequence. Sequencing and bioinformatics were performed on the QIIME2 platform of Shanghai Personal Biotechnology Co., Ltd. (Shanghai, China) and the sequencing results were analyzed based on amplicon sequence variants (ASVs).

### Bioinformatics and statistical analysis

Taxa abundances at the phylum and genus levels were statistically compared between groups. ASV-level alpha diversity indices, Beta diversity Venn diagram and Volcano map were analyzed at the ASVs level. And then, ASVs were aggregated at the genus level, and other results were analyzed at the genus level. Alpha diversity indices, such as Observed species, and Shannon diversity indices were calculated using the ASV table in QIIME2, and visualized as box plots; Beta diversity was evaluated by nonmetric multidimensional scaling (NMDS) based on the Bray–Curtis distance; the significance of differentiation of microbiota structure between groups was assessed by ANOSIM using QIIME2; Volcano map was performed to make a differential analysis of ASVs/genus using R package “DESeq2”; Scores of Linear discriminant analysis effect size(LEfSe), a method for biomarker discovery, measure the consistency of differences in relative abundance between taxa in the groups analyzed (As farmer vs As non-exposed), with a higher score indicating higher consistency. Taxa with linear discriminant analysis scores > 2 and P < 0.05 were considered to be significant. Hierarchical clustering analysis consists of two parts: the left represents the hierarchical clustering tree of community samples, and the right represents the stacked histogram of species composition; UPGMA cluster analysis of Bray–Curtis distance matrix was performed using R package “stat”, and the visualizations were performed using R package “ggtree”; random forest analysis was applied to discriminate the samples from different groups using QIIME2 with default settings. Nested stratified k-fold cross-validation was used for automated hyperparameter optimization and sample prediction; spiral heatmaps were plotted using heatmap tools in the genescloud platform (https://www.genescloud.cn). The data was normalized by z-scores. The package uses popular clustering distances and methods implemented in dist and hclust functions in R.

For the grouped samples (the number of samples in each group ≥ 3), the R package can be used to draw a box line diagram to visually display the differences among different samples in both groups, and the Kruskal–Wallis rank sum test and dunn’s test can be used as post hoc tests to verify the significance of the differences (the Kruskal–Wallis test is equivalent to the Wilcoxon test for both groups of samples). Statistical significance was defined as a P-value < 0.05.

## Results

### Firmicutes were the main bacterial phyla in both farms

**Fig. 1:**
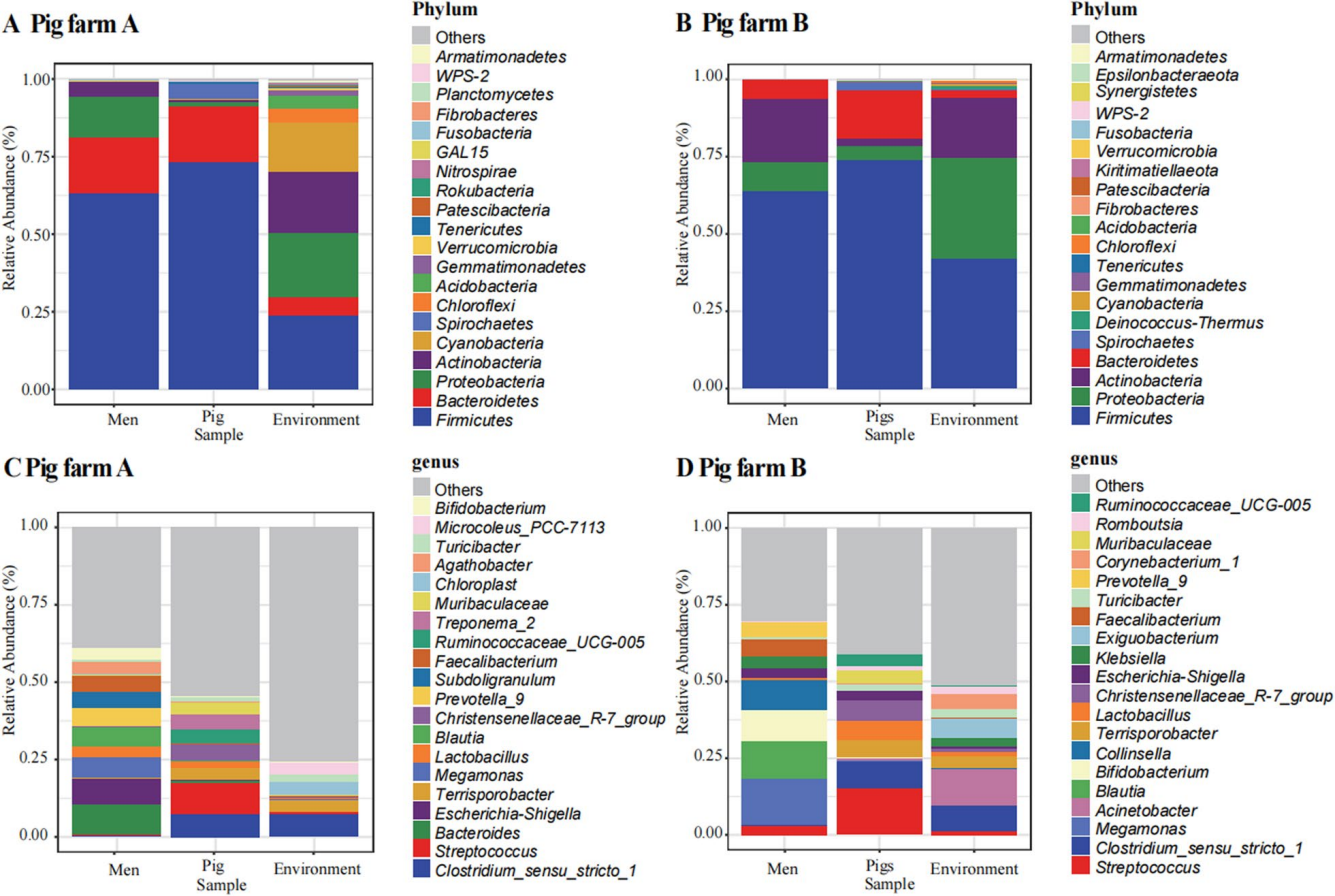
Relative abundance of the sample classification level. **A**, **B** The phylum level of the top 20 phyla in the total sample. **C**, **D** The genus level of the top 20 genera in the total sample

After sequencing analysis, a total of 197,712 ASV sequences were obtained in pig farms. Both farms were divided into two domains, Archaea and Bacteria, by classification annotation. 2081 genera were analyzed in pig farm A while 1196 in pig farms B. Taxa abundances at the phylum and genus levels were statistically compared between groups. Samples were divided into human faeces microbes, pig faeces microbes, and environmental microbes. The taxonomic composition of the total samples was analyzed at the top 20 phylum and the top 20 genus levels (see Fig. 1), as well as 16 phylum and 14 genera were repeated in both farms. Among the top 20 genera, *more Clostridium_sensu_stricto_1* was found in environmental samples and pig faeces microbes (see Additional file 1: Fig. S2 and Additional file 1: Fig S3). The microbial distribution of the two farms showed similar trends.

### The diversity index of Farmer alpha was higher

**Fig. 2:**
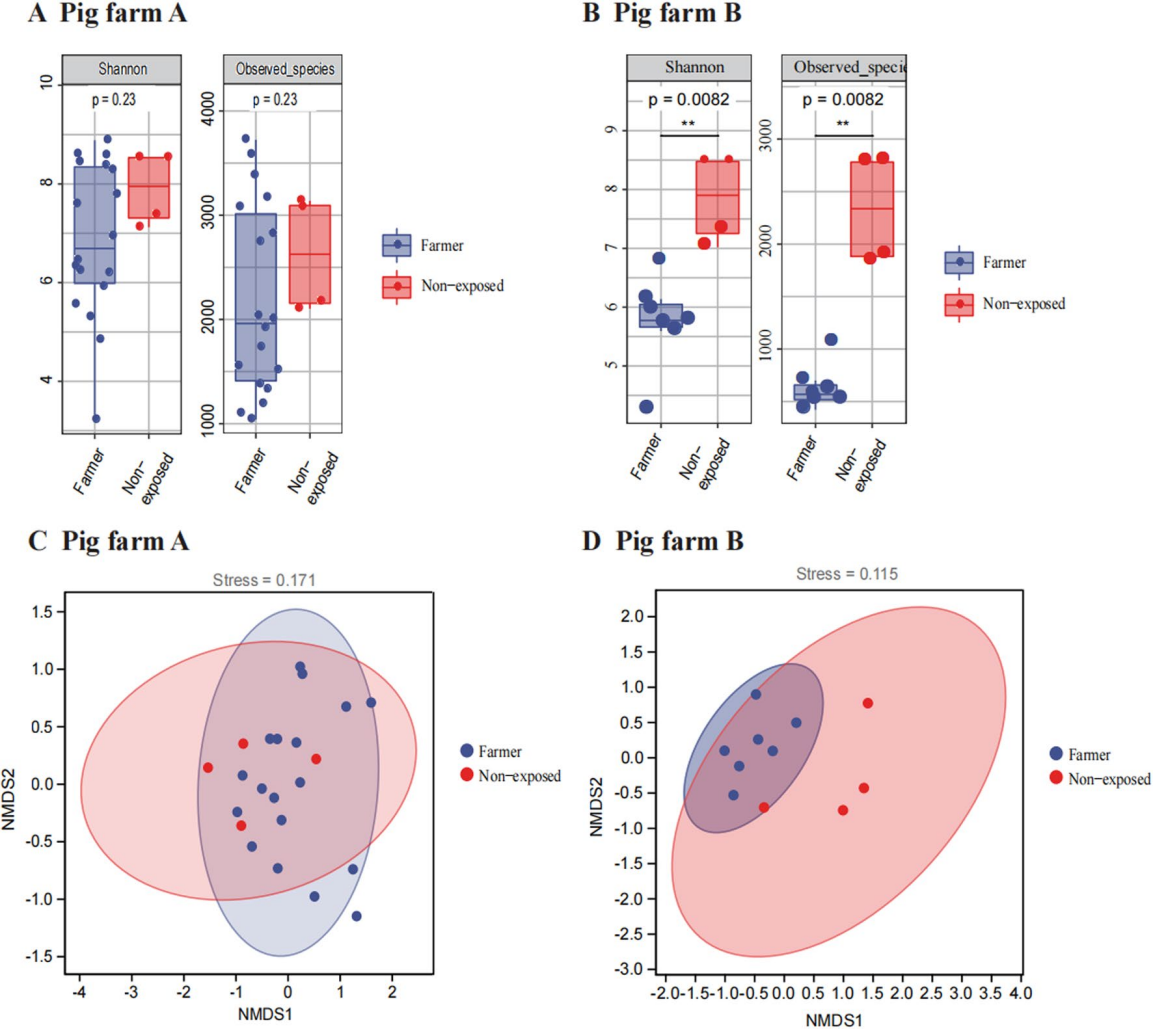
Analysis of human faeces samples from different pig farms: **A**, **B** alpha diversity index of farmers and non-exposed individuals; **C**, **D** Bray–Curtis distance matrix for NMDS analysis;

Human faeces microbe samples were divided into two groups, farmers and non-exposed persons. In terms of alpha diversity indices, non-exposed were slightly higher (see Fig. 2A, B). NMDS analysis was performed using Bray–Curtis distances calculated from the relative abundance of ASVs (see Fig. 2C, D**)**. The results revealed that there was no significant difference between groups of farmers and non-exposed persons in farm A (R = 0.080, *P* = 0.287, ANOSIM). However, a significant difference was observed in farm B (R = 0.245, *P* = 0.027, ANOSIM). The 95% confidence circle indicates that the microbial communities of the farmers are closer, particularly in pig farm B. The Alpha diversity index revealed the same trend of diversity among farmers in both farms.

### Farmers’ faeces microbiota changes with working hours

**Fig. 3:**
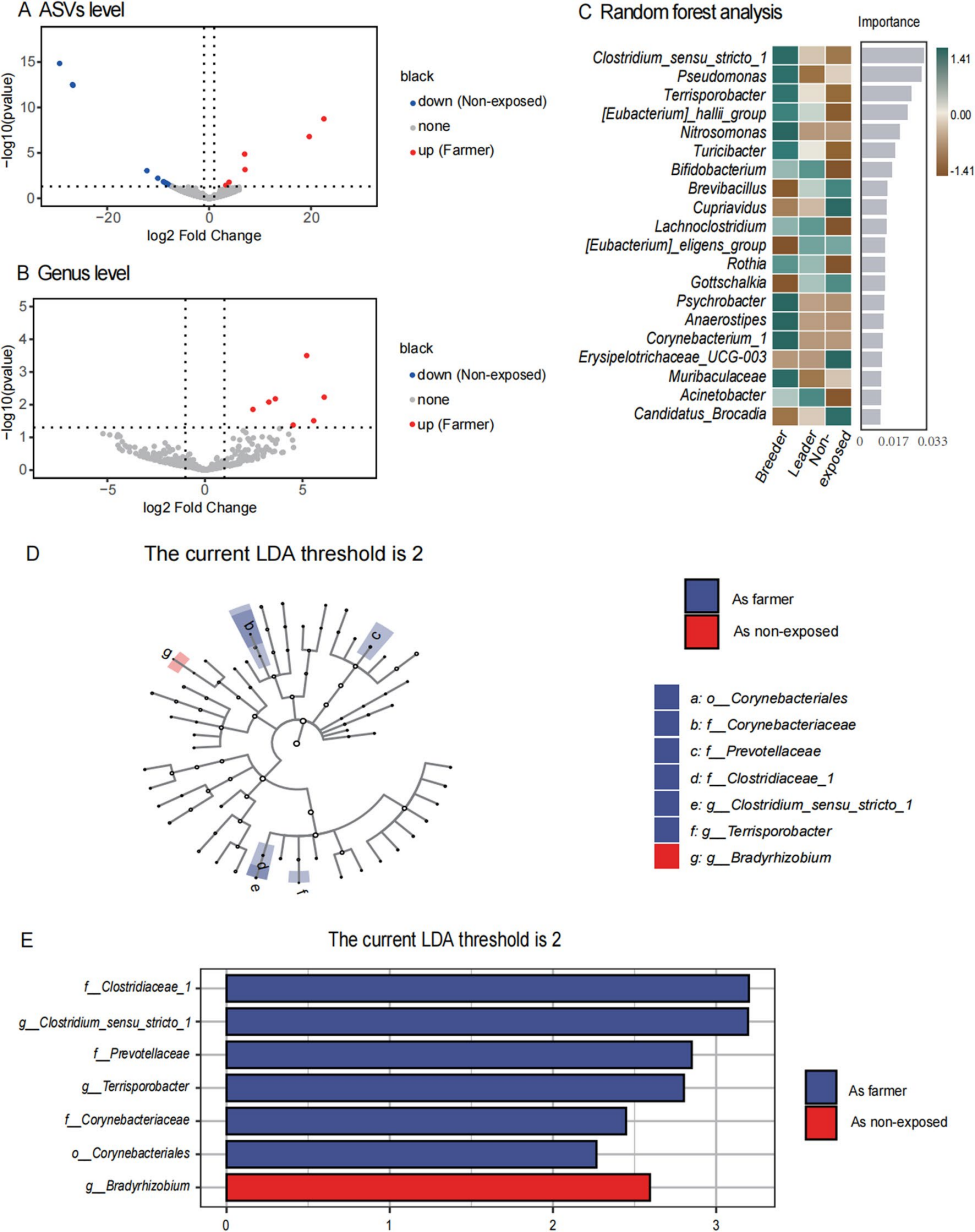
Analysis of faeces samples from all humans: **A**, **B** ASVs level and genera level farmer vs non-exposed volcano diagram. log2FoldChange as abscissa, −  log10 (P-value) as ordinate, horizontal dotted line Y =  −  log10 (0.05), vertical dotted line x =  ± 1. The red dots are marked as 'up' when the log2FoldChange is greater than or equal to 1 and the P-value is less than 0.05. This indicates the enrichment of ASVs in farmers. The blue dots are marked as 'down' when log2FoldChange ≤  −  1 and P-value < 0.05, indicating enriched ASVs in non-exposed persons. No difference is found in gray spots; **C** Random forest analysis of the intra-group mean in human samples. **D**, **E** LEfSe was combined with LDA score to differentiate bacterial biomarker groups biologically and statistically. Bacterial with P < .05 and LDA score > 2 were considered significant

To further explore the effects of pig farming on farmers, samples of all human faeces microbiota were divided into two groups. The volcano maps showed the different ASVs and genera. 6 ASVs are marked as increasing in farmers (see Fig. 3A), including ASV_133662 (*Streptococcus*), ASV_311074 (*Escherichia-Shigella*), ASV_111920(*Terrisporobacter)*, ASV_57963 (*Clostridium_sensu_stricto_1*), ASV_192153(*Faecalibacterium*), and ASV_101783(*Prevotella_9)*. Then, in the volcano map at the genera level (see Fig. 3B), 7 genera were abundant among farmers but not among non-exposed persons, including *Terrisporobacter, Enterococcus, Turicibacter, Clostridium_sensu_stricto_1, Prevotella_7, Nitrosomonas* (see Additional file 2).

In addition, farmers were divided into breeders and leaders based on the duration of their occupational exposure in the production area. Therefore, samples of all human faeces microbes were divided into three groups: breeders’, leaders’ and non-exposed persons’. To get the average value within the group, random forest analysis was performed on all the microorganisms found in human feces (see Fig. 3C). In this random forest analysis, the number of k-fold cross-validations was set to 5. The study found that the 3 genera significantly enriched by farmers, which increased with the increase of exposure time. 4 of the top 20 dominant genera in the Random Forest analysis showed significant enrichment on the genus level volcano map, including *Terrisporobacter, Turicibacter, Clostridium_sensu_stricto_1, Nitrosomonas*. Furthermore, when the number of working hours declines, these genera become less important. It is worth mentioning that 1 of the workers who worked at the pig farms B happened to be one of the non-exposed persons and 2 farmers in pig farm A had been out of work for 3 consecutive months at the time of the second sampling. Therefore, we treated them as non-exposed persons in the second analysis. These 3 people are regarded as "farmer" and "non-exposed persons" according to their working state when they are sampled. *Clostridium_sensu_stricto_1* and *Terrisporobacter* were notably present in the group of farmers (see Fig. 3D and E).

*Clostridium_sensu_stricto_1* and *Terrisporobacter* are frequently mentioned above, and therefore may coexist more easily with humans, yet they exhibit notable differences 3 months after leaving pigs.

### Dust microbes closer to pigs are more similar to pig faeces microbiota

**Fig. 4:**
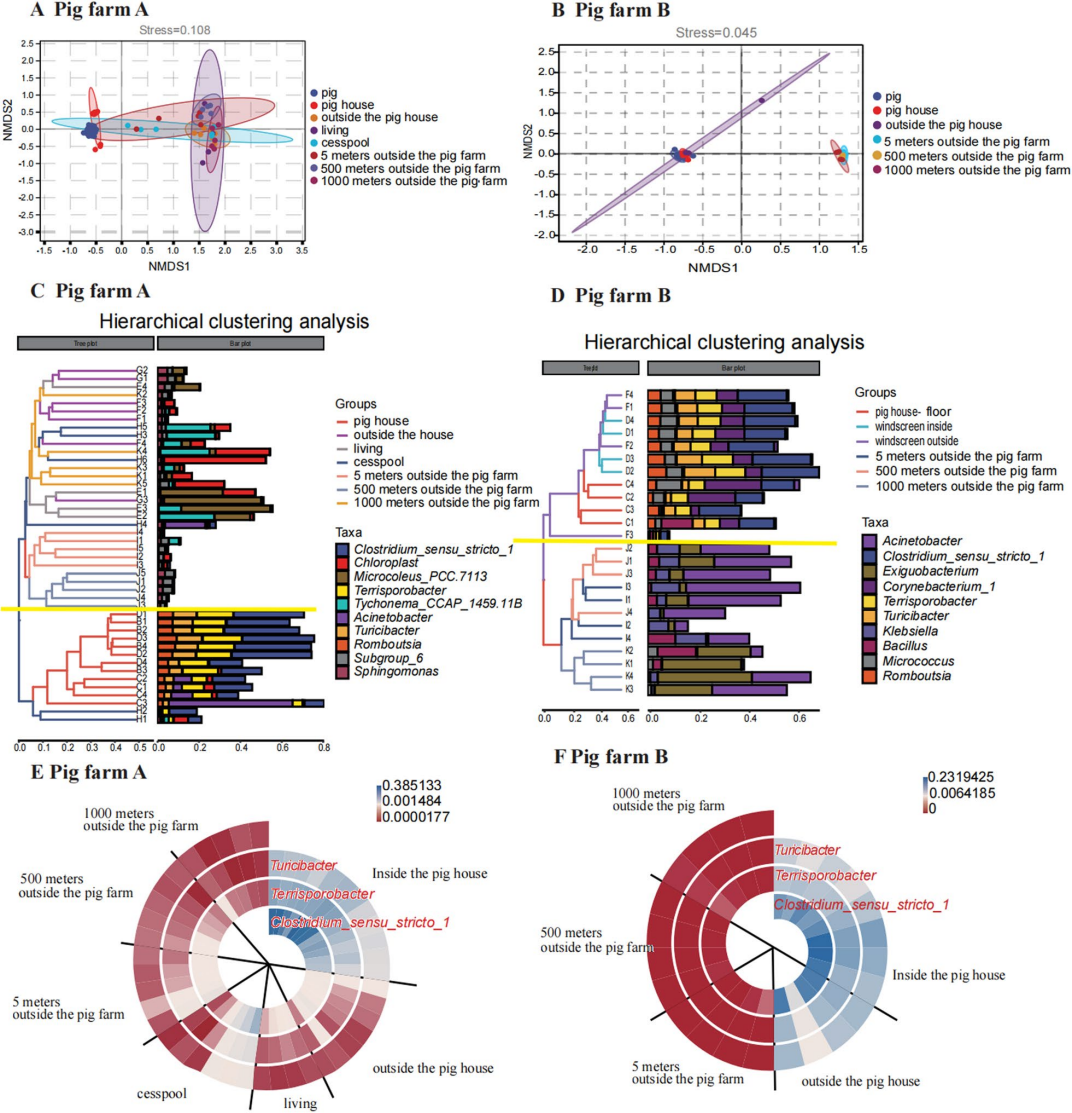
Outdoor environment analysis: **A**, **B** NMDS of environmental samples and pig faeces microbes; **C**, **E** hierarchical clustering analysis of pig farm A environmental dust; **E**, **F** spiral heat map of environmental dust co-occurring genera abundance

Beta diversity analysis was performed on environmental dust and pig faeces microbiota from the two farms. Beta diversity was evaluated by NMDS based on the Bray–Curtis distance. In the NMDS diagram, the pig house dust microbes were closest to the pig faeces microbiota (see Fig. 4A and B). In pig farm A, the study found that the cesspool runs through each group (see Fig. 4A and C). The results of the subsequent anosim test revealed that there was no significant difference between the outside group and cesspool group (*P* = 0.063). Hierarchical cluster analysis of pig farm A shows clustering inside and outside pig house (see Fig. 4C). NMDS diagram and hierarchical cluster analysis of pig farms B shows more obvious clustering inside and outside pig farm (see Fig. 4B and D). In other words, the microbes in the environmental dust outside the farm cluster on a branch.

Therefore, the closer the environmental samples were to pigs, the more distinct species were found. Furthermore, 3 genera can be considered as co-occurring microbes, including *Terrisporobacter* (ASV_111920), *Clostridium_sensu_stricto_1* (ASV_57963), and *Turicibacter* (ASV_20732), which may co-occur between humans and the environment. Therefore, these 3 genera are called the co-occurring microbes in this study.

Environmental samples were drawn and grouped into spiral heat maps using the co-occurring bacteria as a marker (see Fig. 4E and F). The co-occurring microorganisms at the pig farm B, as seen in the figure, were almost nonexistent outside the farm, which may be related to the spray disinfection of the walls and gates there. while the co-occurring microbes at the pig farm A decreased as the distance from the farm increased. The co-occurring microbes detected by Dunn’s test were significantly different inside and 1000 m outside the gate of the pig farm A (see Additional file 1: Fig. S4).

### Simple filtration can reduce cross-infection between pig houses

**Fig. 5:**
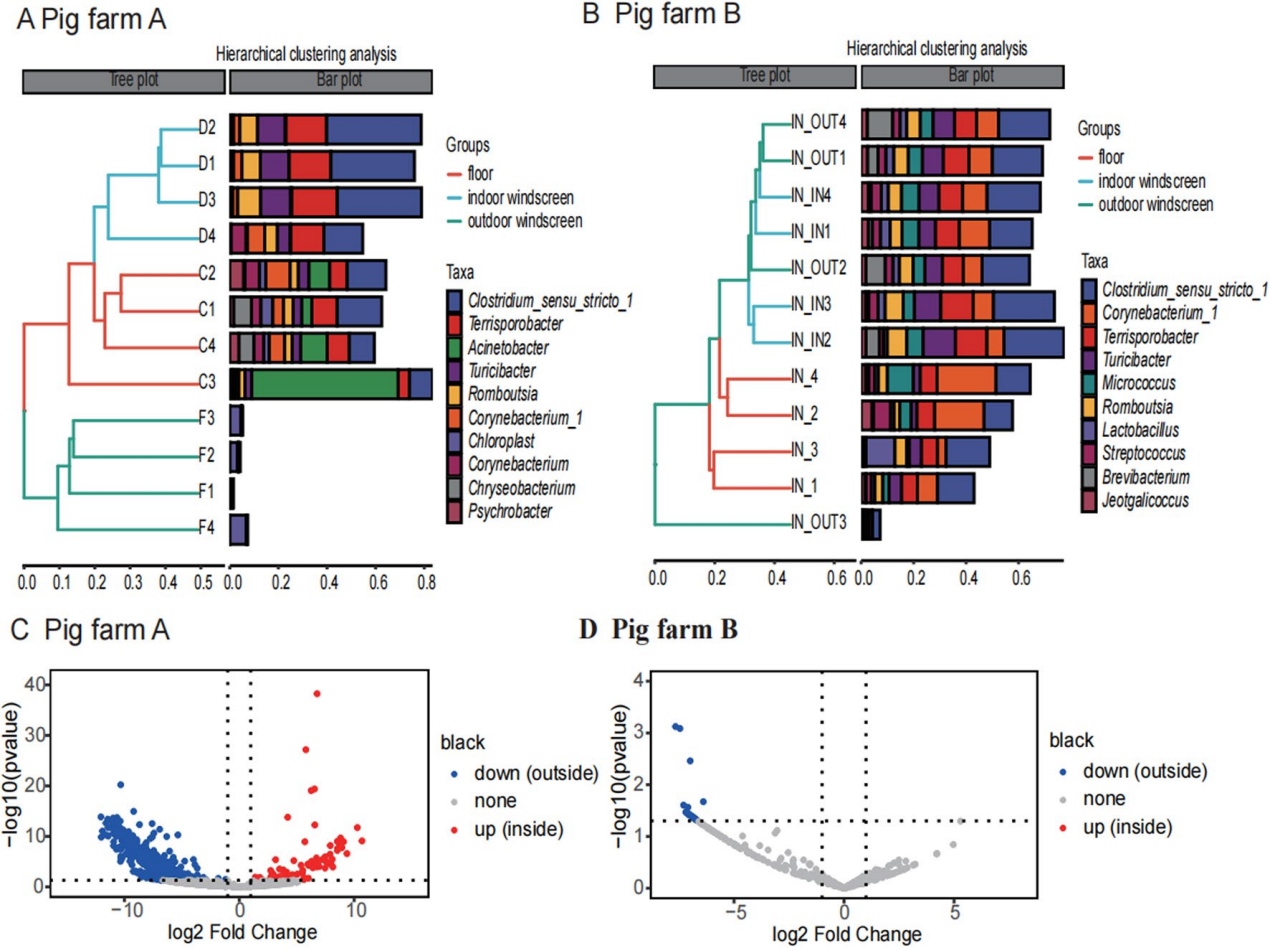
**A**, **B** Hierarchical clustering analysis of environmental microbes; **C**, **D** indoor and outdoor volcano map of windscreen

In the spiral heat maps of the two pig farms, there were significant differences between the inside and outside windscreen of the pig farm A, while there were no significant differences between that of the pig farm B. This was also demonstrated by the cluster analysis diagrams of the floor and inside and outside the windscreen of pig farms A and B, respectively (see Fig. 5A and B). It is known that the pig farm A has a simple filtration system with two screens while the pig farm B has only a simple screen as a filter for exhaust air.

The windscreen inside the pig house was compared with that outside the pig house to identify different microorganisms. A total of 76 genera showed high abundances on the board of the windscreen inside pig farm A, but not in pig farm B (see Fig. 5C and D). The co-occurring microorganisms were all included in the enriched genera (see Additional file 2). This result indicates that in pig farm A neither or less of the co-occurring microbes were able to reach the outside of the house with air. Although microbes can be transmitted by aerosols, simple filtration can reduce cross-infection between houses.

## Discussion

### Effects of the co-occurring microbes on farmers

In the analysis of humans, the study found no significant differences in Observed_species and Shannon index between the two groups, which does not deny that pig farming will result in differences in human faeces microbes. As omnivore, human beings have complex dietary habits. Therefore, the diversity and abundance of human intestinal microbes are in a certain steady state (Martel et al. 2022).

In this study, farmers were found to have lower microbial diversity than non-exposed persons, which is contrary to the results of Kates (Kates et al. 2019) but similar to the result of Vestergaard (Vestergaard et al. 2018). Chinese farms are getting more stringent when it comes to biosafety, and this is shown not just in disinfection and prevention but also in the regulation of farmers’ diets. A pig farm is like a Petri dish with fixed supplies, unifying and fixing farmer’s diet and limiting microbial diversity. This finding could help to explain why farmer samples cluster in the NMDS diagram.

The co-occurring microbes that were prominent on farmers were considered as the three most abundant genera in the environment, which had the highest absolute abundance in pig samples among other samples. On the contrary, the co-occurring microorganisms were less prevalent in areas that were not affected by pigs. In light of this, we hypothesize that the co-occurring microbes came from pigs, and they are susceptible to humans and the environment. According to classification level analysis, the co-occurring microbes, including *Turicibacter*, *Terrisporobacter*, and *Clostridium_sensu_stricto_1,* belong to *Firmicutes*. Short-chain fatty acids are produced and fermented by gut microbes, which accounts for the majority of their physiological contributions. The abundance of *Turicibacter* was related to the digestibility of acid detergent fiber (Niu et al. 2019) and α linolenic acid (X. Gao et al. 2020b). The abundance of *Terrisporobacter* was associated with short-chain fatty acids (Li et al. 2021) and C-reactive protein triglycerides (Lee et al. 2020). The high abundance of *Clostridium_sensu_stricto_1* may have a role in β-oxidation (Usman et al. 2022), which results in the degradation of the majority of long-chain fatty acids. Therefore, it may be inferred that the 3 co-occurring microbes also have an effect on the growth traits of pigs. Xylo-Oligosaccharides (Chen et al. 2021), as a substitute for antibiotics, were found to reduce the relative abundance of *Terrisporobacter* and *Clostridium_sensu_stricto_1* and increase body weight, daily gain, and feed conversion rate in weaned piglets. Furthermore, *Clostridium_sensu_stricto_1* was negatively associated with litter size and daily gain of piglets (R. Wang et al. 2021b; Hu et al. 2021).

The 3 co-occurring microbes in this study are from pathogenic genera, which can cause intestinal inflammation and even cancer and are related to a variety of digestive diseases: *Turicibacter* causes pancreatic cancer (Jeong et al. 2020) and colon cancer (Chung et al. 2021; Wen et al. 2021), and is associated with Parkinson’s disease (Jin et al. 2019) and autoimmune encephalomyelitis (Wang et al. 2021c); *Turicibacter* and *Terrisporobacter* affect type I diabetes (Radwan et al. 2020). Of the 3 co-occurring microbes, researchers are more familiar with the *Clostridium_sensu_stricto_1* class. In addition to being associated with colitis (Yang et al. 2019), fatty liver (Yi et al. 2021), gout (Mendez-Salazar et al. 2021), etc., it is also positively correlated with levels of inflammatory markers (*TNF-α*, *IL-1β*, and *IL-6*) (Yi et al. 2021). Thus, *Clostridium_sensu_stricto_1* is correlated with host inflammatory genes (including *REG3G*, *CCL8*, and *IDO1*) (Wen et al. 2021).

The unexpected aspect of our sampling was the presence of 3 participants, whose identities had been switched between the two groups, making them serve as the control group. Three months after leaving the pig farms, the farms gut microbiota partially reverted to their original microbial composition (Sun et al. 2020). Therefore, we believe those who remained unemployed by the pig farm for 3 months were regarded as the non-exposed group. When the co-occurring microbes in this group of gut flora were examined, it was shown that they had a tendency to decline once out of work. There are significant differences between *Clostridium_sensu_stricto_1* and *Terrisporobacter* (P < 0.05).

### Effects of the co-occurring microbes on the environment

In pig farm A, the 3 co-occurring microbes were significantly less on the windscreen inside the environmental samples taken from the pig house, probably because the windscreen is the furthest away from the pig and is thus harder to touch. The windscreen can detect certain microorganisms that are carried by airborne dust, though. In addition, fewer co-occurring microbes in pig farm A were found on the windscreen outside the pig house. It turns out that this pig farm is in an easy air filtration mode in terms of ventilation, which improves the environmental impact, although no significant differences in the co-occurring microbes inside and outside of the pig house.

After analyzing environmental samples of pig farm B, the environment in the pig house is a branch, and the outside environment is also a branch. Outside of the pig farm, the co-occurring microbes were reduced. Unlike pig farm A, pig farm B just has a layer of gauze for air filtering. Therefore, there weren’t many different bacteria discovered on the windscreens inside and outside the pig house.

A few co-occurring microbes also appeared in the living areas. The microbial community in the living area did not significantly differ from that in other areas of the farm, which may have been caused by the dust present there or carried in by farmers. Because the abundance of co-occurring microbes at the genera level in the living area is the lowest, the environmental pollution caused by farmers can be ignored. Therefore, the results indicate that the co-occurring microbes primarily affect the environment through aerosol diffusion. As is known to all, the co-occurring microbes at the genera level are transmitted in two possible ways, aerosol diffusion or farmers after leaving the farm. Farmers seldom ever carry co-occurring microbes, therefore the co-occurring microorganisms' main source of environmental impact is aerosol diffusion in cesspools or pig houses. Numerous investigations (Ko et al., 2008; White et al., 2019; Moor et al. 2021) have been supported by the findings.

The study found that two samples in cesspool contained several co-occurring microbes, and two samples in cesspool did not contain such co-occurring microbes. The microbial community in the cesspool was seen the greatest difference. Cesspools, especially gut microbes in it, cause huge risks to the environment (Peng et al. 2021) and biological health (Ruang-Areerate et al. 2021; Salem et al. 2021), such as ARG (Van Gompel et al. 2020).

Co-occurring microbes such as *Clostridium_sensu_stricto_1*, *Terrisporobacter*, and *Turicibacter* are non-exposed potential hosts of antibiotic-resistant genes (ARGs) and mobile genetic elements (MGEs) (Q. Wang et al. 2020; J. Wang et al. 2021a). The relative abundance of *tetT* gene (Zhou et al. 2021) was positively correlated with the 3 co-occurring genera (*P* < 0.05).

The ARG transmission can be reduced by filtering bio-aerosols through the pathway (Song et al. 2021b). In addition, compost (Yang et al. 2019) reduces the risk of ARG transmission by reducing the relative abundance of *Clostridium_sensu_stricto_1* and *Terrisporobacter* and antibiotic-resistant genes. The co-occurring mibrobe is also used by humans to participate in some fermentation work. *Terrisporobacter* and *Clostridium_sensu_stricto_1* participate in anaerobic digestion (Detman et al. 2021; Song et al. 2021a). More narrowly, *Terrisporobacter* is a main microorganism during Xiaoqu wine fermentation (Su et al. 2020) and *Clostridium_sensu_stricto_1* is abundant in glucose fermentation broth (Lu et al. 2020).

At 1000 m outside the pig farm A, the environmental pollution in the pig house is not significant. Environmental contamination brought on by co-occurring microbes has been lessened when aerosols move through ventilation and filtering systems. In pig farm B, the 3 co-occurring microbes showed the same trend: the farther away from the gate of the pig farm, the fewer co-occurring microbes there are. In the study of pig farms, the impact of distance from porcine gut microbes might be steadily diminished by disinfection. Although microbes can be transmitted by aerosols, simple filtration can reduce cross-infection between houses.

In conclusion, the study found that the microbes *Turicibacter, Terrisporobacter*, and *Clostridium_sensu_stricto_1* were more prevalent in pig breeders and their abundance increased with longer working hours. Furthermore, these microbes were also found in greater quantities in areas closer to the pigs. Further analysis showed that *Clostridium_sensu_stricto_1* and *Terrisporobacter* significantly decreased after 3 months of leaving the pig farm. Additionally, the abundance of these 3 microbes considerably dropped at a distance of 1000 m from the pig farm. The implementation of a simple filtration system can effectively reduce the cross-infection of these 3 microbes between pig houses.

### Supplementary Information

**Additional file 1: Figure S1.** Photo of Windscreen. **Figure S2.** In A pig farm: The top 20 genera of pigs were analysed. **Figure S3.** In B pig farm: The top 20 genera of pigs were analysed. **Figure S4**. In pig farm A: The three co-occurring genera detected by Dunn’s test were significantly different.

**Additional file 2:** Volcano diagram table.

## Data Availability

The raw sequencing data in this study were deposited in the NCBI Sequence Read Archive (SRA) under accession number PRJNA984441.
