# Supplementary material for: Effects of microbes in pig farms on occupational exposed persons and the environment
Source: AMB Express. 2023 Nov 30;13:136. doi: 10.1186/s13568-023-01631-x (PMC10689614; doi:10.1186/s13568-023-01631-x)
Supplement: Supplementary file 1 — Additional file 1: Figure S1. Photo of Windscreen. Figure S2. In A pig farm: The top 20 genera of pigs were analysed. Figure S3. In B pig farm: The top 20 genera of pigs were analysed. Figure S4. In pig farm A: The three co-occurring genera detected by Dunn’s test were significantly different. [file 13568_2023_1631_MOESM1_ESM.docx]

# Additional Material

# Supplementary Figures


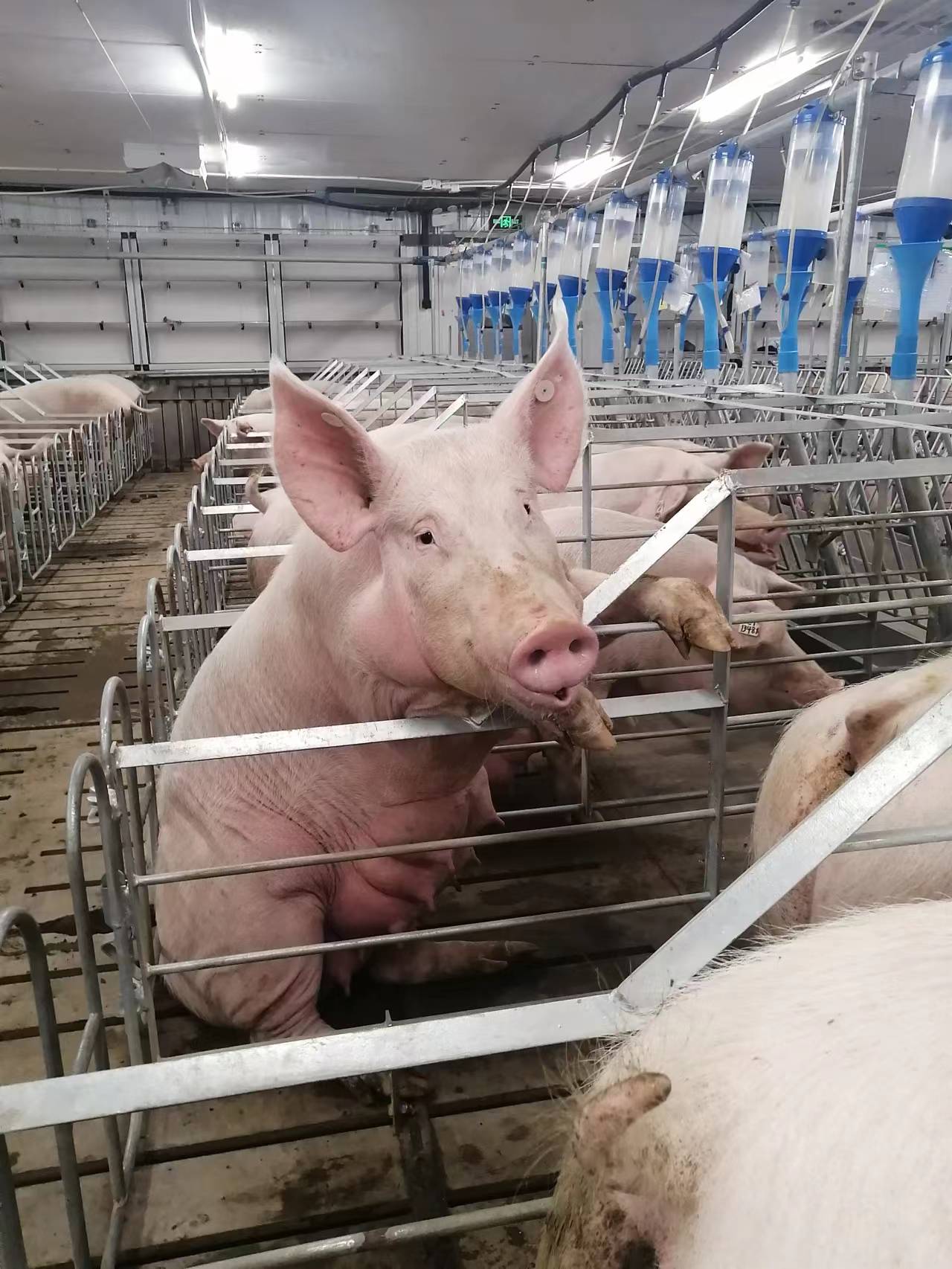


Windscreen: A deflector plate used to regulate the airflow in a pig house.

Additional file 1: Figure S1. Photo of Windscreen

Windscreen: A deflector plate used to regulate the airflow in a pig house. Samples were collected both inside and outside the piggery.

**
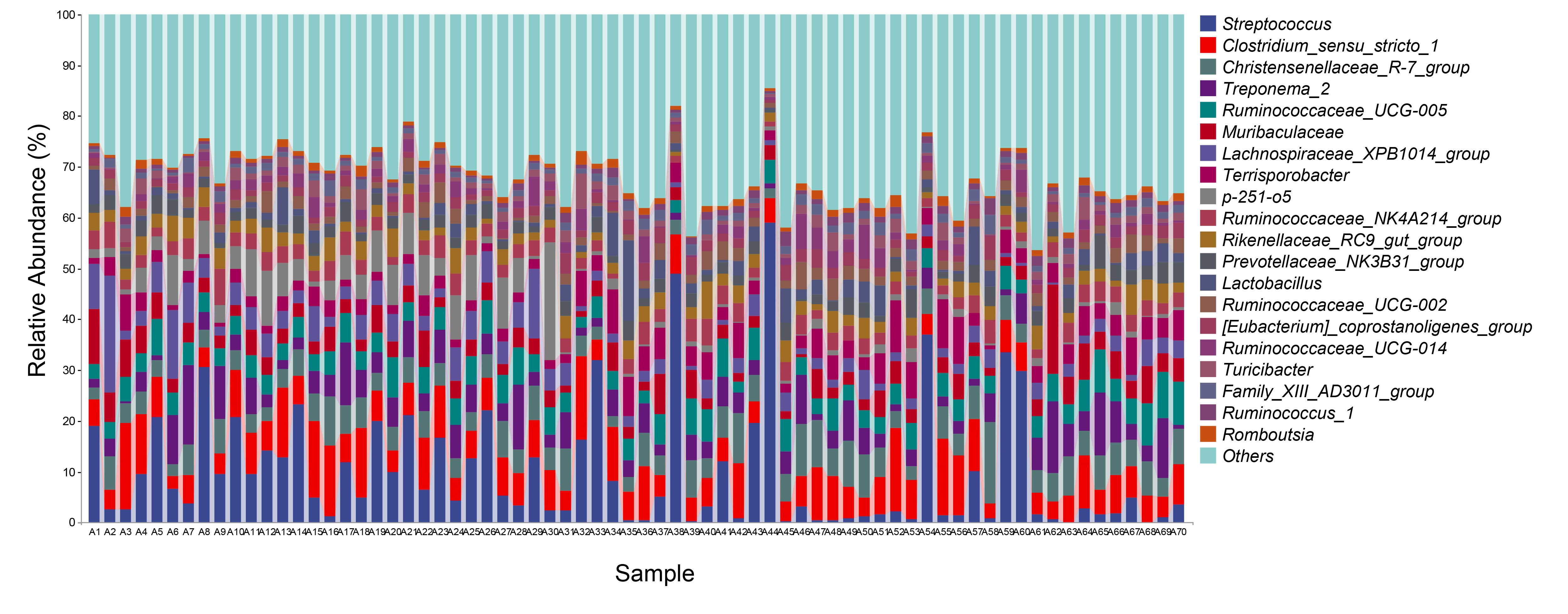
**

Additional file 1: Figure S2. In **A pig farm: The top 20 genera of pigs were analyzed**

**
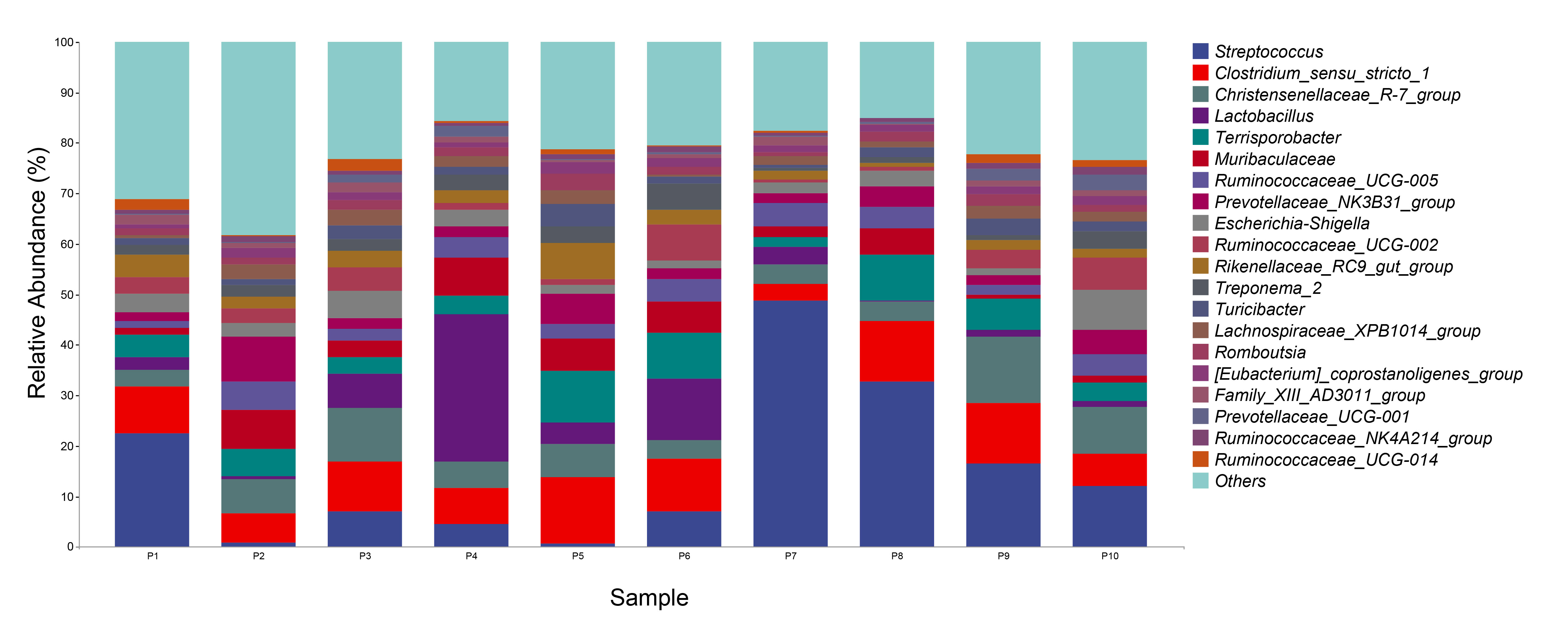
**

A**dditional file 1: Figure S3. In B pig farm: The top 20 genera of pigs were analyzed**





Additional file 1: Figure S4 In pig farm A: The three co-occurring genera detected by Dunn’s test were significantly different.
